# A Dynamic Multi-Scale Feature Fusion Network for Enhanced SAR Ship Detection

**DOI:** 10.3390/s25165194

**Published:** 2025-08-21

**Authors:** Rui Cao, Jianghua Sui

**Affiliations:** Navigation and Ship Engineering College, Dalian Ocean University, Dalian 116023, China; caorui_dlou@163.com

**Keywords:** SAR ship detection, CSP_DTB, DYDDH, YOLOv11, RepGFPN

## Abstract

This study aims to develop an enhanced YOLO algorithm to improve the ship detection performance of synthetic aperture radar (SAR) in complex marine environments. Current SAR ship detection methods face numerous challenges in complex sea conditions, including environmental interference, false detection, and multi-scale changes in detection targets. To address these issues, this study adopts a technical solution that combines multi-level feature fusion with a dynamic detection mechanism. First, a cross-stage partial dynamic channel transformer module (CSP_DTB) was designed, which combines the transformer architecture with a convolutional neural network to replace the last two C3k2 layers in the YOLOv11n main network, thereby enhancing the model’s feature extraction capabilities. Second, a general dynamic feature pyramid network (RepGFPN) was introduced to reconstruct the neck network architecture, enabling more efficient multi-scale feature fusion and information propagation. Additionally, a lightweight dynamic decoupled dual-alignment head (DYDDH) was constructed to enhance the collaborative performance of localization and classification tasks through task-specific feature decoupling. Experimental results show that the proposed DRGD-YOLO algorithm achieves significant performance improvements. On the HRSID dataset, the algorithm achieves an average precision (mAP50) of 93.1% at an IoU threshold of 0.50 and an mAP50–95 of 69.2% over the IoU threshold range of 0.50–0.95. Compared to the baseline YOLOv11n algorithm, the proposed method improves mAP50 and mAP50–95 by 3.3% and 4.6%, respectively. The proposed DRGD-YOLO algorithm not only significantly improves the accuracy and robustness of synthetic aperture radar (SAR) ship detection but also demonstrates broad application potential in fields such as maritime surveillance, fisheries management, and maritime safety monitoring, providing technical support for the development of intelligent marine monitoring technology.

## 1. Introduction

As an active remote sensing method, synthetic aperture radar (SAR) can be mounted on multiple aerial platforms, including aircraft, satellites, and spacecraft. The technology facilitates uninterrupted, weather-resilient Earth surface surveillance while possessing limited penetration abilities through surface substances.

SAR is distinguished from conventional radar by its higher resolution, capability to function in any weather conditions, and resistance to variations in illumination [1,2,3]. Therefore, SAR is extensively utilized for automatic ship detection in maritime operations, including defense and security, monitoring of fishing vessels, and oversight and rescue efforts in maritime transportation [4,5]. The application of SAR-based ship image detection in maritime surveillance has become increasingly prevalent and crucial. Traditional SAR ship detection methods are primarily based on Constant False Alarm Rate (CFAR) and its associated algorithms [6,7]. It mainly relies on the difference in scattering intensity between the target and the sea clutter to realize detection. However, when facing the texture refinement of sea clutter brought by high-resolution SAR imaging, the multipath scattering effect in complex marine environments, and the diversity of scattering characteristics of different types of ships, the CFAR method shows obvious limitations in environmental adaptability and insufficient target generalization capability, leading to performance degradation, with increasing false alarm rates and decreasing detection rates in complex application scenarios.

In contemporary research, owing to the steady advancement in machine learning, detection algorithms employing deep learning have found extensive use in the SAR ship detection domain. Object detection algorithms employing deep learning are generally structured into two major categories: one-stage target detection algorithms and two-stage target detection algorithms. Two-stage detection algorithms, such as R-CNN [8], Fast R-CNN [9], and Faster R-CNN [10], achieve high-precision detection through a cascade process involving region proposal and fine classification. However, they face a bottleneck in computational efficiency. In contrast, one-stage target detection algorithms, represented by You Only Look Once (YOLO) [11] and Single Shot Multi-Box Detector (SSD) [12], excel in real-time performance but often sacrifice accuracy. The academic community has proposed a number of improvements to optimize the performance of the first-stage YOLO object detection algorithm. Cong Li, et al. [13] solved the problems of low detection accuracy and high false negative rate for small and dense targets in SAR ship images through the channel feature enhancement module (CFE) and feature selection fusion network (FSFN). Tang, H. et al. [14] optimized feature extraction and fusion using multi-scale receptive field attention mechanism convolutional blocks (AMMRF), solving the problems of low detection accuracy and high computational resource consumption in SAR image ship target detection caused by diverse target sizes, complex backgrounds, and redundant parameters in traditional models. Tang, X. et al. [15] combined deformable convolutional networks (DCNets), dynamic sparse attention mechanisms (BiFormer), and Wise IoU loss functions to propose the DBW-YOLO method, which solves the problems of false negatives and false positives in the detection of coastal and small vessel targets in SAR images in complex environments. Wu, K. et al. [16] combined an improved CFAR detection algorithm (MCFAR), a coordinate attention mechanism (CA), and a progressive feature fusion module (AFF) to solve the problems of false negatives and false positives in ship detection in SAR images caused by complex backgrounds and multi-scale targets. The work of the above scholars has, to a certain extent, promoted the advancement of SAR ship image recognition research, but there are also the following shortcomings:

1. The detection method improvements proposed by the above scholars mainly focus on optimizing false positives and false negatives in ship detection tasks caused by multi-scale detection in SAR images. Therefore, when processing a large number of small targets, detection accuracy may be affected.
2. Most of the improvements made by the above scholars focused on improving accuracy without giving sufficient consideration to parameter issues. Although Wu, K. et al. [16] took parameter issues into account, the parameters still reached 5.855 M.

To counter these challenges, this work presents an optimized YOLOv11n algorithm called DRGD-YOLO (YOLOv11n combined with CSP_DTB, RepGFPN, and DYDDH). The main contributions of this paper are as follows:

1. To address the issues of false positives and false negatives in SAR ship image target detection tasks caused by multi-scale changes in ships and a large number of small targets, this paper proposes the DRGD-YOLO detection algorithm, which achieves higher detection accuracy while balancing parameters.
2. A hybrid structure, CSP_DTB, was designed to replace the last two layers of C3k2 in the main trunk, expand the receptive field of the model, reduce the loss of small target de-pendency information caused by an insufficient receptive field, and enhance the feature extraction ability of the model. Compared with existing traditional transformer-based methods, our main innovation lies in the design of a dynamic transformer block (DTB), which dynamically allocates feature channels to convolutional neural networks (CNNs) and transformer branches through learnable channel ratio parameters (tcr) to achieve complementary processing. It also combines the convolutional gated linear unit (CGLU) to replace standard feedforward networks, thereby reducing the computational cost of transformers while maintaining global modeling capabilities.
3. A Dynamic Decoupled Dual-Alignment Head was designed to replace the detection head of YOLOv11, enhancing information interaction between the classification and localization decoupling heads and facilitating superior localization and classification in the detection head. The main novelty of the DYDDH module lies in its use of a task decomposition mechanism guided by layer attention, which dynamically reconstructs convolution kernel parameters through weights generated by adaptive global average pooling to achieve feature decoupling for classification and regression tasks. It also combines a spatial adaptive alignment mechanism and classification probability attention to perform task-specific feature alignment. Compared to traditional channel or spatial attention mechanisms, our method dynamically modulates at the convolution weight level, enabling more precise handling of the differences in feature requirements and spatial misalignment issues between classification and localization tasks in SAR target detection.

## 2. Methods

### 2.1. Introduction to the YOLOv11 Algorithm

The YOLOv11 algorithm is an object detection algorithm launched by Ultralytics (Frederick, MD, USA) on 30 September 2024. Compared to its previous generations, the main architecture of YOLOv11 consists of three key components: the backbone network, the neck network, and the detection head. The main innovation lies in the introduction of the C3k2 module, which replaces the traditional C2f module. The C3k2 module adopts a dual-branch small kernel convolution design, optimizing the computational efficiency of cross-stage partial connections by decomposing a single large convolution into two parallel small convolution operations. This structure retains the feature fusion advantages of the CSP Bottleneck while significantly reducing parameter complexity and computational overhead. The improved module enables more efficient multi-scale feature extraction capabilities in YOLOv11.

YOLOv11 incorporates the C2PSA module in its backbone network. The C2PSA module adopts a cross-stage partial connection and a spatial attention fusion design. Through the integration of a spatial attention mechanism into the cross-stage feature aggregation process, it dynamically enhances the feature response of key areas. While retaining the gradient optimization advantages of the CSP structure, it introduces an adaptive spatial weight distribution mechanism, enabling the network to focus more on areas with high semantic information. The improved structure significantly enhances detection accuracy in complex scenarios in YOLOv11, particularly demonstrating stronger feature recognition capabilities in small object identification and dense object discrimination tasks.

**Figure 1:**
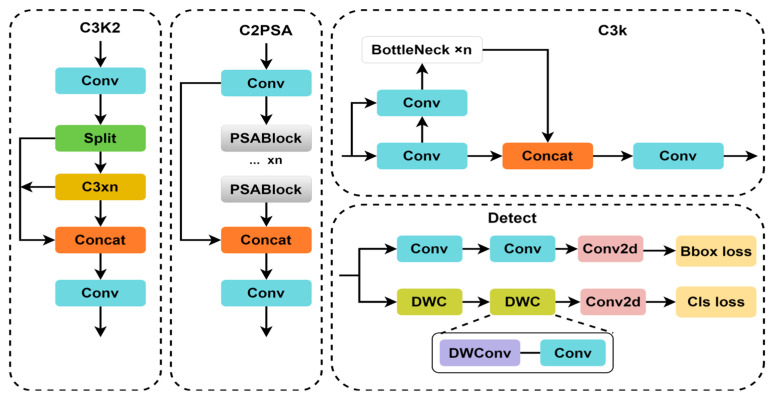
Improved module architecture of the YOLOv11 network.

In terms of detection head design, YOLOv11 continues to adopt a dual decoupled head design for classification and localization. The main innovation lies in the use of smaller convolution kernels in the design of the classification decoupled detection head, which improves computing efficiency while reducing computing parameters. The improved module architecture of the YOLOv11 network is shown in Figure 1.

### 2.2. Improved DRGD-YOLO

**Figure 2:**
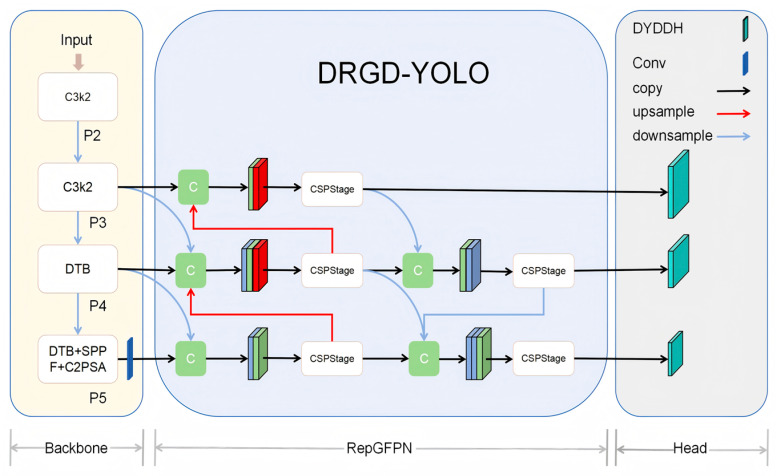
DRGD-YOLO structure diagram. The model uses a hybrid backbone network constructed with modules such as CSP_DTB and C3k2 to extract multi-scale features. It achieves bidirectional cross-scale feature fusion through RepGFPN, and finally, the DYDDH detection head performs dynamic task decoupling and alignment to output detection results for three scales: P3, P4, and P5.

Ultralytics’ experimental results showed that the YOLOv11 series algorithms exhibited higher performance than previous versions. Although YOLOv11 has demonstrated excellent performance for object detection tasks, further improvements can be achieved in SAR ship image detection. This paper builds upon YOLOv11n as the base model for improvement and proposes a SAR ship image detection model called DRGD-YOLO, whose structure is shown in Figure 2.

### 2.3. Cross-Level Partial Dynamic Transformer Block

In the field of object detection, Transformer models have garnered significant attention due to their powerful global feature extraction capabilities. In contrast, the YOLO series of algorithms based on traditional convolutional neural network (CNN) [17] architectures have a limited receptive field. This limitation restricts them to extracting only local features during the feature extraction process, leading to the loss of dependent information for small objects and resulting in false detections and missed detections of small objects. In SAR ship detection, a large number of small objects are typically involved. Wang et al. [18] pointed out that expanding the limited region is an effective method to improve detection performance. Meanwhile, the self-attention mechanism in Transformers can effectively enhance the model’s global representation capability on feature maps, enabling large-scale feature extraction and effectively alleviating the issue of detectors being constrained by small-scale perceptual regions. Therefore, this study proposes integrating the multi-head self-attention mechanism (MHSA) [19] from the Transformer architecture into the model to expand its perceptual region. However, the significant computational complexity introduced by the Transformer architecture poses challenges; directly applying the Transformer across all channels not only increases the number of parameters but also elevates the computational load, despite potentially improving detection accuracy. To address this challenge, this study proposes a hybrid architecture termed CSP-DTB, which combines Transformer modules with convolutional neural networks (CNNs). The overall design of CSP-DTB adopts a cross-stage partitioning (CSP) [20] structure, dividing the input into two parts: one part for skip connections and the other processed through dynamic Transformer blocks (DTB). DTB divides the input features into two parts based on a predefined Transformer channel ratio (TCR): one part is processed through residual blocks in the CNN branch, and the other part is processed through multi-activation convolutional gated linear units (MA-CGLU) in the Transformer branch. The channel division follows:

(1) $$C_{t}=\left[C\times TCR\right],C_{c}=C-C_{t}$$

The processed features are merged using a fusion function:

(2) $$Y_{-}fused=Conv_{1\times 1}\left(Concat\left(\left[Y_{-}cnn,Y_{-}transformer\right],dim=1\right)\right),$$

The MA-CGLU module combines the multi-head self-attention (MHSA) mechanism with convolutional gated linear units (CGLU) [21] and adopts a residual structure in its design:

(3) $$X_{1}=X+DropPath\left(MHSA\left({LayerNorm}_{1}\left(X\right)\right)\right),$$

(4) $$X_{2}=X_{1}+DropPath\left(CGLU\left({LayerNorm}_{2}\left(X_{1}\right)\right)\right),$$

The CGLU operation is defined as follows:

(5) $$CGLU\left(X\right)=Conv\left(X\right)⊙\sigma \left(Conv\left(X\right)\right),$$

**Figure 3:**
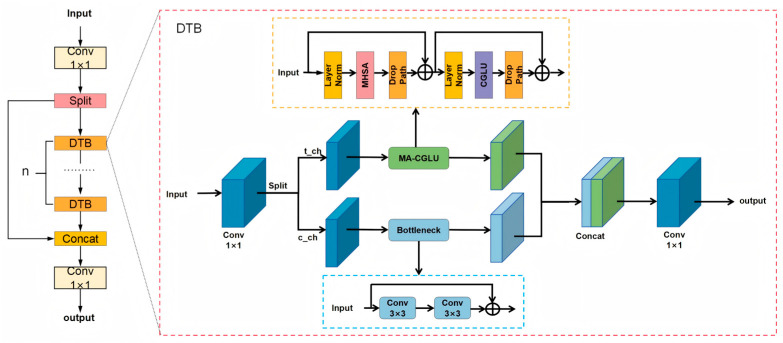
CSP_DTB structure diagram. The CSP_DTB module adopts a CSP structure and divides the input features into two paths through 1 × 1 convolution: One path bypasses directly, while the other path passes through a cascaded DTB module. Subsequently, these two paths are concatenated and compressed through 1 × 1 convolution to output fusion features.

Multi-head Self-Attention (MHSA) enhances the model’s ability to capture overall semantic information by analyzing the correlations between distant pixels in an image. The Convolutional Gated Linear Unit (CGLU) module improves the nonlinear representation of features through the synergistic design of convolutional operations and gating mechanisms, dynamically adjusting the information transmission weights between feature channels to effectively filter key features and suppress redundant noise. By leveraging the MHSA module and CGLU module, this paper develops an efficient feature processing architecture. MHSA provides the model with rich contextual information from a global perspective, helping CGLU better integrate global semantic information when processing local features. Meanwhile, CGLU’s precise screening and enhancement of local features provide MHSA with a more accurate feature set. Additionally, the CSP-DTB module adjusts the number of channels used for the Transformer based on configurable channel ratio parameters (TCR), enabling performance and efficiency to be balanced according to different model sizes and computational resource constraints. The CSP_DTB structure diagram is shown in Figure 3.

### 2.4. Utilizing RepGFPN to Replace the Neck

YOLOv11 features a Path Aggregation Feature Pyramid Network (PAFPN) [22] structure in its neck. The PAFPN structure is an improved multi-scale fusion structure based on the FPN structure. Its primary principle is to enhance bidirectional information exchange between low-level and high-level features by introducing bottom-up paths. Specifically, the PAFPN structure builds upon the top-down paths of the FPN structure by adding additional bottom-up lateral connections. This allows semantic information from low-resolution feature maps to be fused with adjacent high-resolution feature maps through upsampling, while the localization details of lower-level features are propagated to deeper layers of the network through downsampling. This bidirectional path design enables more effective transmission of feature information across different levels, thereby improving the model’s localization accuracy for multi-scale targets.

**Figure 4:**
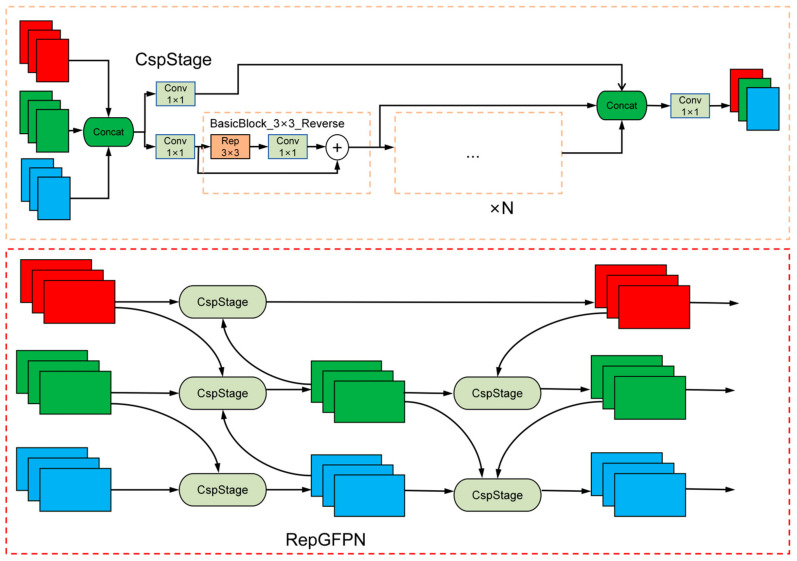
Structural diagram of RepGFPN. RepGFPN performs bidirectional fusion and cross-layer aggregation on P3–P5 features and finally outputs optimized features through structural reparameterization.

Nevertheless, this paper finds that the PAFPN structure still has certain limitations when processing SAR ship image detection tasks. PAFPN primarily focuses on feature interaction between adjacent layers during feature fusion, but it lacks direct fusion of features from the shallower P2 and P3 layers. In SAR ship detection tasks, which involve a large number of small targets, the detailed information from shallow-layer features is critical for detection. Therefore, the structural design of PAFP may result in the gradual loss of texture information from small targets in deeper network layers. With the aim of addressing this problem, this paper introduces the RepGFPN, as proposed by Xu et al. [23], to reconstruct the neck network of YOLOv11, promoting the exchange of large-scale and small-scale feature information and enhancing the model’s feature fusion capabilities. RepGFPN modifies the GFPN [24] structure by integrating multiscale features into the horizontal features of both the previous and current layers. Simultaneously, it achieves more efficient information transfer through cross-layer connections, allowing information to be conveyed to deeper network layers. The primary principle involves eliminating the redundant up-sampling operation in the GFPN post-fusion. Additionally, CSPNet is utilized in the feature fusion block to replace the original feature fusion method based on 3 × 3 convolution. This approach is further improved by incorporating a reparameterization mechanism and connecting efficient layers (ELAN). As a result of these enhancements, the model’s feature fusion capability is significantly improved. The structural diagram of RepGFPN is shown in Figure 4.

### 2.5. Dynamic Decoupled Dual-Alignment Head

**Figure 5:**
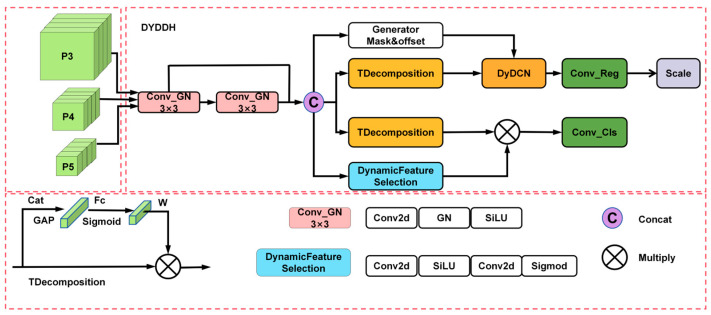
The structure of DYDDH. After extracting basic features through shared convolution, the DYDDH module uses a task-decoupled structure (dynamic weight adjustment) to separate the classification and regression branches, perform spatial attention modulation and deformation convolution alignment separately, and finally fuse the prediction results to output detection boxes and category probabilities.

The target detection decoupling head of YOLOv11 typically utilizes independent classification and localization branches, which are responsible for predicting the location and species of the target, respectively. This independent design results in a lack of effective information exchange between the two branches, which may reduce the accuracy of target localization in complex detection scenarios and increase the likelihood of missed or incorrect detections. Additionally, this decoupled head design exhibits certain limitations when addressing multi-scale variations in targets. The lack of information interaction between classification and localization can lead to missed detections, particularly of small targets, during multi-scale changes, which usually involve a large number of small targets and multi-scale changes in the SAR ship detection task. This paper draws inspiration from the concepts proposed by Feng et al. [25] in TOOD and has developed a Dynamic Decoupled Dual-Alignment Head (DYDDH) to enhance task interaction between the two detection heads—localization and classification. The structure of this design is illustrated in Figure 5.

The detection head consists of four key modules: a shared feature extraction module, a task decomposition module, a dynamic alignment module, and a localization classification prediction generation module. These modules work together to provide efficient feature extraction and accurate target detection. First, the detection head extracts initial features from the input features through shared convolutional layers. Then, the task decomposition module decouples the features for classification and localization tasks. Subsequently, dynamic alignment processing is applied to the classification features and localization features separately. Finally, the corresponding localization prediction convolutional layers and classification prediction convolutional layers are generated, and all values are concatenated to obtain the detection results described in this paper.

In the shared convolution module, shared convolution differs from the batch normalization (BN) [26] convolution used in previous studies. In this paper, we replace BN convolution with group normalization (GN) convolution based on the idea of GN [27]. The idea of GN can be expressed as follows:

(6) $${\hat{x}}_{i}=\frac{x_{i}-\mu_{g}}{\sqrt{\sigma_{g}^{2}+\epsilon }}\cdot \gamma +\beta ,$$

In this context, $\mu_{i}^{g}$ and $\sigma_{i}^{g}$ denote the mean and standard deviation of the features in group g, γ and β represent the learnable scaling parameter and offset parameter, respectively. Unlike BN, which only considers the batch dimension, GN groups data in the channel dimension, making it more stable during mini-batch training. Compared to BN, GN demonstrates higher performance and better generalization capabilities. In the FCOS [28] paper, GN has been proven to improve the localization and classification performance of detection heads. Additionally, by using shared convolutions, not only can computational costs be reduced, but high-quality interaction features can also be provided for subsequent task decomposition modules.

**Figure 6:**
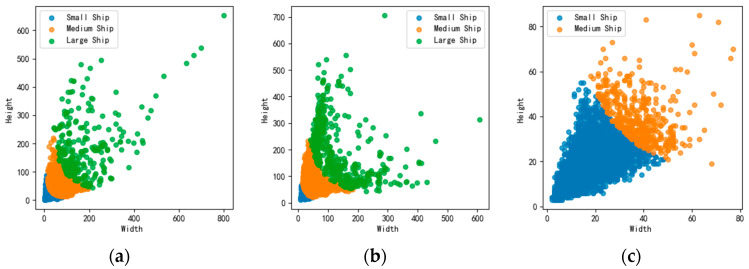
Ship size distribution: (**a**) HRSID; (**b**) SSDD; (**c**) LS-SSDD.

In the task decomposition module, considering that the two different tasks of localization and classification share the same features, which may lead to conflicts, this paper uses the task decomposition module (TDecomposition) to generate hierarchical weights through the attention mechanism and dynamically calculate the interaction features between tasks, thereby promoting the decomposition of localization and classification tasks. The module first obtains global context information through global average pooling, then calculates attention weights through a two-layer bottleneck structure and finally achieves task-specific feature decomposition through weighted feature fusion. The TDecomposition module structure diagram is shown in Figure 6.

In the dynamic alignment module, to improve the localization capability of localization features, this paper utilizes deformable convolution [29] and generates masks and offsets through interaction features. The generated offsets and masks allow the convolution kernel to dynamically adjust the sampling position on the feature map, thereby more accurately capturing the geometric details of the target. The principle of deformable convolution can be understood as follows: For the input feature X,

(7) $$y\left(p\right)=\sum_{k=1}^{K}w_{k}\cdot x\left(p+p_{k}+\Delta p_{k}\right)\cdot \Delta m_{k},$$

where $p_{k}$ denotes the sample point offset of the standard convolution, $\Delta p_{k}$ represents the learned additional offset, $\Delta m_{k}$ indicates the modulation factor that controls the weight of each sample point, and $W_{k}$ signifies the convolution kernel weights.

In the classification branch, this paper uses probabilistic convolutions to generate spatial attention weights, enabling dynamic feature selection. Classification features are obtained by element-wise multiplication with the probabilistic weights to yield the final features.

Finally, this paper separately processes classification features and localization features to generate corresponding localization prediction convolutions and classification prediction convolutions. To address the issue of inconsistent target scales detected by each detection head, this paper uses a learnable Scale layer to scale features while employing shared convolutions, thereby balancing multi-scale feature responses. All values are concatenated to obtain the detection results of this paper.

## 3. Experiments and Discussions

### 3.1. Experimental Environment and Configuration

The experimental environment configuration used in this study includes the Ubuntu operating system, an Intel (R) Xeon (R) Gold 5418Y 10-core central processing unit, and an NVIDIA RTX 3090 graphics processing unit (Santa Clara, CA, USA). The deep learning framework used is PyTorch 2.0.0, with CUDA version 11.7, and the programming language is Python 3.10. During training, the modified DRGD-YOLO network did not load any pre-trained model weights. When the loss function reached a stable state, the model training was considered complete. The specific hyperparameter information of the model used during training is detailed in Table 1.

### 3.2. Introduction to the Dataset

The HRSID [30] public dataset, the SSDD public dataset [31], and the LS-SSDD-v1.0 public dataset [32] have been selected for this analysis. The HRSID dataset was released by the University of Electronic Science and Technology in 2020 and is specifically designed for SAR ship detection missions, serving as a high-quality benchmark dataset. It contains 5604 high-resolution SAR images, with an average of three ship targets per image, totaling 16,951 instances. The SSDD dataset was produced by the Department of Electronics and Information Engineering at the Naval Aeronautics and Space University specifically for the ship detection task, and it contains a total of 1160 images and 2456 ships, with an average number of ships per image of 2.12. The LS-SSDD-v1.0 dataset was constructed by Prof. Xiaoling Zhang’s team at the University of Electronic Science and Technology, generating 9000 slice-level samples through spatial slicing and manual labeling, with a total of 6015 ship targets labeled. The target name of the dataset is “ship.” When dividing the dataset, HRSID and SSDD are allocated in a 7:1:2 ratio for the training set, validation set, and test set, respectively, while LS-SSDD-v1.0 is divided into a training set and validation set in an 8:2 ratio. The HRSID dataset serves as the main dataset, and the model’s ablation experiments and the comparative experiments of different modules are carried out on this dataset. SSDD and LS-SSDD-v1.0 are mainly used to validate the generalization ability of the model, and the comparison experiments of the model are also conducted on SSDD and LS-SSDD-v1.0. The dataset is divided into three categories of vessels based on their pixel area size: small vessels (pixel area less than 482 pixels), medium vessels (pixel area between 482 and 1452 pixels), and large vessels (pixel area greater than 1452 pixels) [30,31,32]. The scatter plots showing the distribution of the three vessel sizes in the three datasets are shown in Figure 6.

### 3.3. The Evaluation Indicators of the Experiment

In order to evaluate the efficacy of the model, this research employs four principal metrics: Precision, Recall, F1 Score, and Average Precision (AP). Precision (P) is defined as the ratio of accurately identified positive cases to the total number of instances classified as positive, thereby serving as an indicator of the model’s predictive accuracy. In contrast, Recall (R) assesses the proportion of true positive samples that the model successfully identifies in relation to all actual positive cases, thereby reflecting the model’s sensitivity to positive instances. While Precision provides insight into the reliability of positive predictions, Recall offers an assessment of the model’s ability to encompass positive samples. The mathematical formulations for these metrics are presented as follows:

(8) $$Precision=\frac{TP}{TP+FP},$$

(9) $$Recall=\frac{TP}{TP+FN},$$

In this context, TP (true positives) denotes the targets that have been accurately identified, FP (false positives) signifies the targets that have been incorrectly identified, and FN (false negatives) pertains to the actual targets that the model has failed to detect. Average Precision (AP) is determined as the mean precision across various levels of recall for a specific target. When precision (P) is plotted against recall (R), the resulting graph is referred to as the precision-recall (P-R) curve. The Average Precision is represented by the area under this curve, which is computed using the following integral formula:

(10) $$AP=\int_{0}^{1}P\left(R\right)dR,$$

The Average Precision (AP) serves as a comprehensive evaluation metric that assesses model performance by computing precision values across the full spectrum of Intersection over Union (IoU) thresholds at different recall levels. IoU quantifies the spatial overlap ratio between predicted and ground truth bounding boxes, functioning as the primary criterion for distinguishing True Positive (TP) detections from False Positive (FP) instances. The IoU computation follows the mathematical relationship:

(11) $$IoU=\frac{A\cap B}{A\cup B},$$

where A represents the prediction frame of the model, which is also known as the detection frame and B represents the true frame, which is considered as TP when IoU ≥ threshold; otherwise, it is considered as FP. The meanings of AP50:95, AP50, and AP75 used in this paper are shown in Table 2.

MAP is the average of multiple targets and is calculated as follows:

(12) $$mAP=\frac{1}{N}\sum_{i=1}^{N}AP_{i}=\int_{0}^{1}P\left(R\right)dR,$$

N represents the target type. Since only one type of target is detected in this paper, N = 1. At this point, the mean Average Precision (mAP) is equal to the Average Precision (AP). In addition to the AP metrics, we also introduce the F1 score to evaluate the model. The F1 score is another important metric for measuring model performance, as it balances precision (P) and recall (R). It is formulated as follows:

With these metrics, we can conduct a comprehensive evaluation of the model.

(13) $$F_{1}=2\cdot \frac{precision\cdot recall}{precision+recall},$$

To comprehensively assess the real-time detection capability of the proposed model, this study evaluated three key performance metrics: frames per second (FPS) for processing speed, model parameters for computational complexity, and floating-point operations per second (FLOPs) for computational efficiency. FPS is defined as follows:

(14) FPS=1/T

where *T* represents the detection time required for processing a single image. FPS denotes the number of images that can be processed per second on the designated test platform, specifically referring to the average frame rate achieved across the validation dataset. Both model parameters and FLOPs serve as indicators of the model’s computational complexity.

### 3.4. Ablation Experiment

To assess the effectiveness of individual components introduced in this work, we maintained consistent experimental parameters while systematically implementing various module configurations alongside a baseline architecture. Comprehensive ablation studies were performed on the HRSID dataset, with optimal performance metrics highlighted in bold formatting. The quantitative outcomes of these ablation analyses are presented in Table 3.

**Figure 7:**
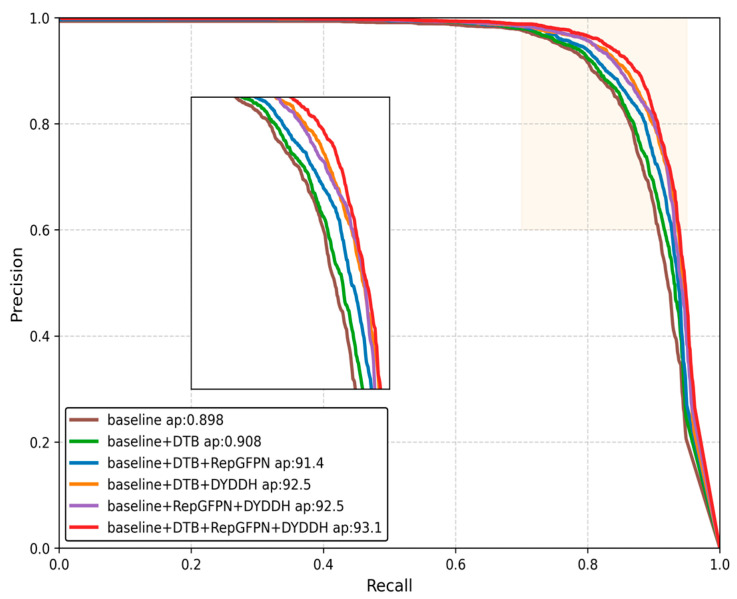
Precision–recall maps for ablation experiments.

Figure 7 shows a mainstream performance comparison of the YOLO series. From the image, it can be seen that different modules are introduced one by one based on the baseline, and the coverage area of the curve increases accordingly. When the two modules designed in this paper and the RepGFPN module are combined, the curve occupies a larger area and has a wider coverage range, which also verifies the correctness and rationality of the method proposed in this paper.

### 3.5. Comparative Experiments

To experimentally validate the effectiveness of the various modules and algorithm improvements, this paper conducted comparative experiments introducing different module improvement strategies and corresponding algorithm comparison experiments on the HRSID dataset. At the same time, to ensure the comprehensiveness of the comparative experiments, this paper also included other detection algorithms for comparison. The corresponding comparative experiments are as follows.

#### 3.5.1. Comparison of Various Classical Pyramid Network Structures

To substantiate the effectiveness of introducing the RepGFPN feature pyramid structure, this paper conducts comparative experiments by introducing several common pyramid structures. The experimental comparison data are tabulated in Table 4. Among them, BIFPN is a bidirectional feature pyramid network, while GDFPN is based on the concept of global information fusion, proposing a new collection and distribution mechanism (GD) on the basis of GFPN to convolve basic blocks and attention basic blocks for feature information extraction and fusion. HSFPN is a multi-level feature fusion pyramid. The experimental results clearly show that the RepGFPN structure achieves the highest metrics in mAP50, mAP75, F1, and mAP50–95. Although it also introduces higher parameters, this paper believes that the higher accuracy metrics justify the parameter trade-off. In summary, the introduction of the RepGFPN structure brings significant benefits to the model.

#### 3.5.2. Comparison of the Effects of Different Detection Heads

To systematically measure the performance of the detection head proposed in this paper, we conducted comparative experiments on the HRSID dataset by introducing several mainstream detection heads. This paper uses YOLOv11n as the baseline and sequentially replaces the detection heads with SEAMHead, PGI, MultiSEAMHead, EfficientHead, and the DYDDH designed in this paper for comparison experiments. Among these, SEAMHead and MultiSEAMHead are detection heads integrated with attention mechanisms, PGI is improved by adding programmable gradient information proposed in YOLOv9, and EfficientHead is a lightweight detection head. The obtained results are tabulated in Table 5.

The experimental results demonstrate that the detection head designed in this paper achieved the highest scores in mAP50 and F1, while also having the fewest parameters. Although its performance in mAP75 and mAP50:95 was slightly lower than that of PGI and MultiSEAMHead, the latter two also had more parameters. Overall, the detection head designed in this paper outperformed other mainstream detection heads.

#### 3.5.3. TCR Sensitivity Analysis in CSP_DTB

In this paper, the TCR parameters in the CSP_DTB module are set to 0.25 and 0.5 for Layer6 and Layer8, respectively. To validate this parameter selection, we conducted a sensitivity analysis using five combinations: (0.25, 0.25), (0.25, 0.5), (0.25, 0.75), (0.5, 0.5), and (0.75, 0.5). This design evaluates the individual effects of each parameter while using (0.25, 0.5) as the baseline configuration. All experiments maintained identical conditions except for the TCR parameter settings, employing the same dataset and evaluation metrics. The experimental results are shown in Table 6.

The results of the sensitivity analysis validated the effectiveness of the selected TCR parameters. When the TCR parameters for Layer6 and Layer8 were set to 0.25 and 0.5, respectively, the model achieved optimal performance across three key metrics: mAP50 (93.1%), F1 (88.9%), and mAP50:95 (69.8%). In contrast, while the parameter combination (0.25, 0.25) performs similarly on some metrics, the model’s parameter count increases to 2.98 M, which is 0.03 M more than the selected combination. Other parameter combinations exhibit varying degrees of performance degradation across all metrics. Therefore, the TCR parameter combination adopted in this study achieves both detection accuracy and good parameter efficiency, demonstrating the rationality of the parameter selection.

#### 3.5.4. Model Generalization, Validation, and Comparison Test

To demonstrate the efficacy of the enhanced algorithm introduced in this work, comprehensive comparative evaluations were performed on the HRSID dataset. The parameter settings for the comparative experiments are shown in Table 7. All experimental results were obtained under the same conditions.

The experimental framework incorporated several established mainstream approaches for benchmarking purposes, including Faster-RCNN, RTMDet-tiny, YOLOx-tiny, and Cascade-RCNN. Given the increased parameter count in the enhanced algorithm, our analysis encompassed not only comparisons with compact variants of the same methodology but also integrated the S variant from the YOLO family as a reference baseline. The quantitative findings from these evaluations are documented in Table 8.

Based on the experimental findings presented herein, it is evident that while the proposed methodology exhibits higher parameter complexity compared to the baseline approach on the HRSID dataset, our algorithm maintains substantially fewer parameters than the S variant within the same algorithmic family, while achieving comparable accuracy performance. Notably, our method achieves superior performance across key evaluation metrics, securing top rankings in Precision (P), Recall (R), mAP50, and F1 score. In conclusion, the algorithmic enhancement strategy introduced in this work demonstrates considerable effectiveness and practical viability.

**Figure 8:**
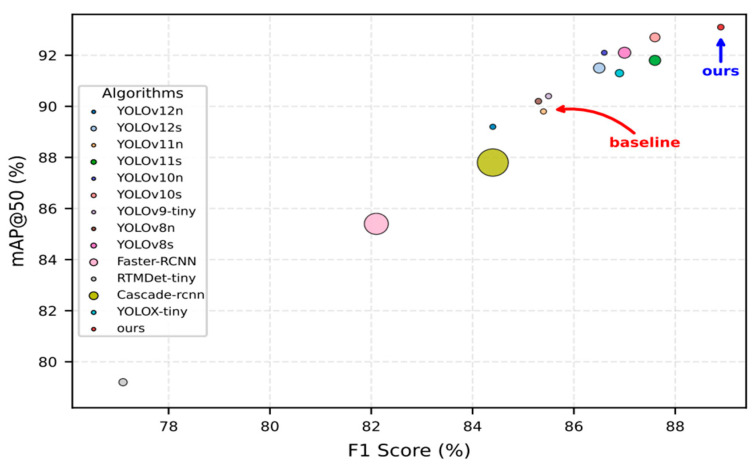
Performance comparison on the HRSID dataset.

To provide a more intuitive visualization of the comparative experimental outcomes on the HRSID dataset, a bubble chart representation is employed to illustrate the performance analysis. The horizontal axis corresponds to F1 Score values, while the vertical axis represents mAP50 metrics. The bubble dimensions indicate the respective model parameter magnitudes. Figure 8 presents the algorithmic performance comparison visualization for the HRSID dataset.

To assess the generalization capability of the proposed methodology, additional validation was conducted using the SSDD and LS-SSDD-v1.0 datasets through comparative evaluations, enabling simultaneous performance assessment and generalization verification. The quantitative outcomes are documented in Table 9.

The experimental findings indicate that the developed model demonstrates superior generalization capabilities. Across both evaluation datasets, all performance indicators exhibit enhancement relative to the baseline architecture (YOLOv11n). For the LS-SSDD-v1.0 dataset, improvements were observed as follows: mAP50 increased by 1.5%, mAP75 enhanced by 1.1%, mAP50:95 advanced by 0.4%, and F1 Score elevated by 1.4%. Similarly, on the SSDD dataset, performance gains included: mAP50 advancement of 0.7%, mAP75 enhancement of 1.7%, mAP50:95 improvement of 3.6%, and F1 Score increase of 1.2%. Remarkably, within the n-series algorithmic family, our enhanced methodology achieves superior performance across all evaluation criteria and exceeds the majority of s-series approaches. When benchmarked against alternative algorithmic frameworks, our proposed technique exhibits marginally lower performance than Cascade-RCNN on the LS-SSDD-v1.0 dataset, yet maintains merely 1/20th of Cascade-RCNN’s parameter complexity. On the SSDD dataset, our enhanced approach sustains optimal performance across all four evaluation metrics.

In conclusion, the algorithmic enhancement strategy presented in this work demonstrates rationality and robust generalization performance.

## 4. Visualization of the Effects of Model Improvement

### 4.1. Comparison of Inference Result Visualization

For visual confirmation of the model’s superior performance, three representative images were extracted from the HRSID, SSDD, and LS-SSDD-v1.0 datasets, and inference verification was then performed using the YOLOv11n and DRGD-YOLO models. The inference results are shown in the figure. Green represents correctly detected objects, orange represents false positives, and red represents missed detections.

**Figure 9:**
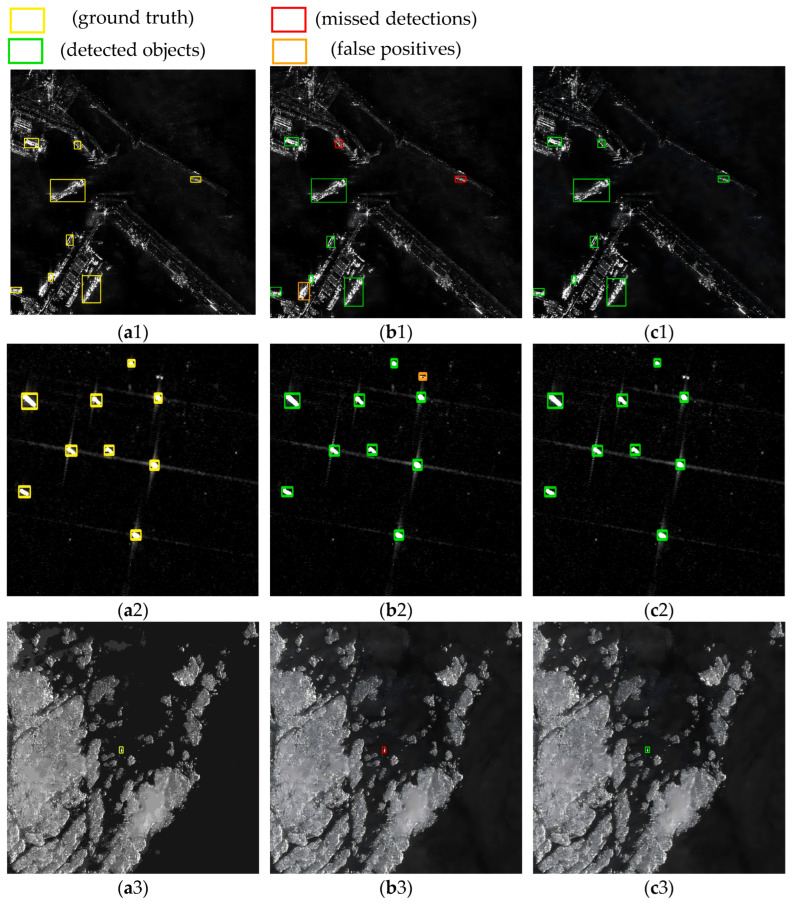
Inference visualization results on the HRSID, LS-SSDD-v1.0, and SSDD datasets. (**a1**–**a3**) shows the ground truth for HRSID, LS-SSDD-v1.0, and SSDD, (**b1**–**b3**) shows the inference results for YOLOv11n, and (**c1**–**c3**) shows the inference results for DRGD-YOLO.

Figure 9(a1–a3) illustrates the ground truth, while Figure 9(b1–b3,c1–c3) presents the inferred results of YOLOv11 and DRGD-YOLO, respectively. In Figure 9(a1), the background depicts a near-coastal scene where the ocean meets the land, characterized by a complex and cluttered environment. The detection targets consist of multi-scale ships, including large, medium, and small vessels. It is evident that YOLOv11 fails to detect two small ships and generates one false alarm, whereas DRGD-YOLO successfully identifies all targets and minimizes false alarms. In Figure 9(a2), the detection background primarily features the ocean with slight trailing noise interference. Here, YOLOv11 produces one false alarm, while DRGD-YOLO effectively reduces false alarms and detects all targets. Figure 9(a3) presents a detection scenario where an island reef meets the ocean, which poses additional challenges due to the complexity of the detection environment and increased interference. In this scenario, YOLOv11 fails to detect a ship, while DRGD-YOLO successfully identifies the target without generating false alarms or detections. In summary, the visual inference of DRGD-YOLO across the three datasets demonstrates a significant improvement in performance compared to the original YOLOv11 model, highlighting the effectiveness and robustness of the algorithm enhancements.

### 4.2. Heat Map Visualization Comparison

To verify whether the features learned by the model align with the expectations outlined in this paper and to detect any potential cheating behavior during the learning process, this study employs heatmaps to visualize the gradient calculation results of the model within the images. Gradient-Weighted Class Activation Map (Grad-CAM) [40] played a crucial role in this process, enabling the paper to accurately pinpoint the model’s attention focus during image processing and thereby intuitively determine whether the model effectively captured key features closely related to the task. This paper utilized Grad-CAM to perform feature gradient visualization on SAR ship images from three datasets, visually demonstrating the model’s decision-making basis and its relevance to the ship detection task. The intensity of red regions in the heatmap directly reflects the model’s attention to local features; the more prominent the red, the stronger the concentration of feature extraction.

**Figure 10:**
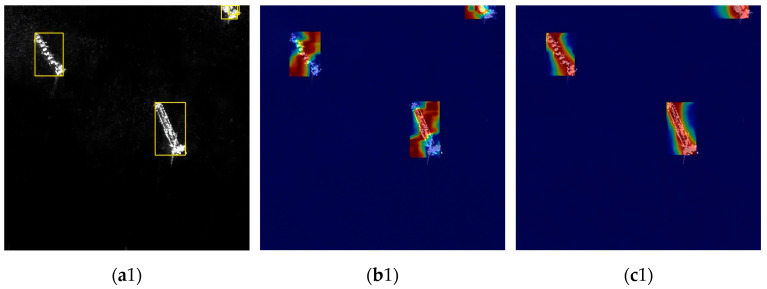

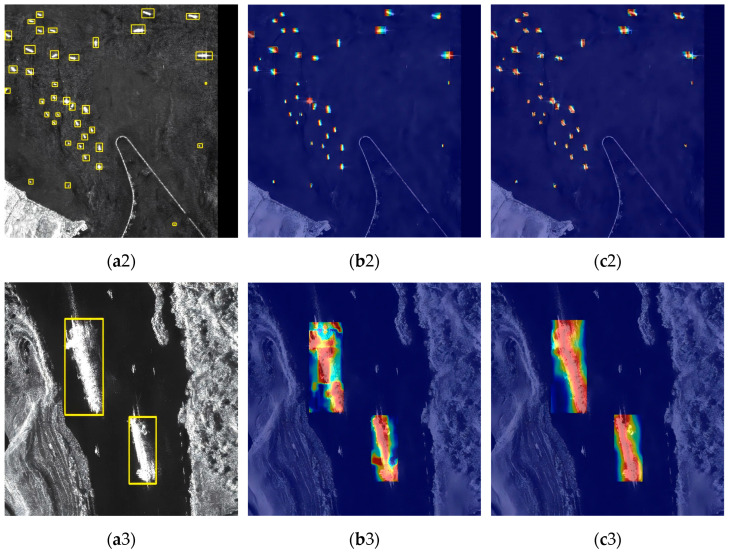
Heat map visualization comparison on the HRSID, LS-SSDD-v1.0, and SSDD datasets. (**a1**–**a3**) shows the ground truth for HRSID, LS-SSDD-v1.0, and SSDD, (**b1**–**b3**) shows the inference results for YOLOv11n, and (**c1**–**c3**) shows the inference results for DRGD-YOLO.

Figure 10(a1) illustrates the offshore samples from the HRSID dataset. It is evident that YOLOv11 emphasizes the ship, with some highlighted areas deviating from the target object. In contrast, GRGD-YOLO focuses more directly on the target itself, with the generated red areas more accurately corresponding to the ship. Figure 10(a2) features samples from the near-coastal region of the LS-SSDD dataset, which contains a significant number of small targets. The heatmap produced by YOLOv11 reveals a greater number of non-red areas, indicating that YOLOv11 struggles to recognize these small targets. Conversely, GRGD-YOLO exhibits markedly better performance, with the generated red areas consistently covering the samples. Figure 10(a3) is selected from the SSDD dataset’s coastal samples, primarily focusing on two large vessels. It can be observed that DRGD-YOLO remains highly focused on the vessels themselves during detection, while YOLOv11 remains relatively dispersed. This demonstrates that the improved method proposed in this paper, GRGD-YOLO, exhibits superior performance in addressing multi-scale vessel detection tasks. In summary, through the visualization of heatmaps, this paper demonstrates that the proposed model possesses:

(1) Enhanced Feature Focus Capability: The GRGD-YOLO model demonstrates a superior ability to concentrate on features that are directly relevant to the target. For instance, in the selected HRSID samples, the GRGD-YOLO model directs greater attention to the ship itself, whereas the attention of YOLOv11 is comparatively more dispersed.
(2) Excellent Detection of Small Targets: In the selected LS-SSDD samples, the DRGD-YOLO model demonstrates superior performance, with its generated thermograms covering a significant portion of the target area. This indicates that the improved model is more adept at capturing relevant information.
(3) Superior Multi-Scale Detection Performance: The DRGD-YOLO model demonstrates a strong focus on the ship targets themselves, rather than on irrelevant details, even when applied to the selected large target samples from the SSDD dataset. This indicates that the DRGD-YOLO model exhibits superior performance in addressing the challenges of multi-scale SAR ship image detection tasks.

## 5. Discussion

In addition to detection accuracy, the computational efficiency of the detection network is also a critical factor. Table 10 compares the performance of the baseline network YOLOv11n with the method proposed in this paper in terms of computational efficiency and inference time. To improve the model’s detection accuracy for SAR ship targets, we increased the model’s complexity and the resources required for network training, which resulted in higher accuracy improvements in this paper but inevitably led to an increase in inference time consumption. However, this paper believes that the increase in inference time consumption remains within a controllable range.

The model proposed in this paper demonstrates excellent detection performance on three datasets; however, it still has limitations. Although we achieved higher detection accuracy, the total number of model parameters increased from 2.55 million to 2.95 million, and the computational complexity rose from 6.3 GFLOPs to 10.4 GFLOPs, resulting in a corresponding decrease in inference speed. This poses challenges for real-time edge deployment of the model. In future research, we will consider the following aspects: First, although the datasets used in this paper include complex detection scenarios, they do not account for extreme environmental conditions, such as extreme weather. Future plans include noise processing of the datasets, adding rain and fog scenarios, and strengthening collaboration with maritime authorities to collect more comprehensive datasets to validate the robustness of the proposed model under extreme conditions. Second, given the real-time requirements of SAR ship detection, a balance must be struck between network complexity and computational resource consumption. Although our model achieves higher detection performance, future research will explore lightweight modifications to the network architecture, such as removing redundant channels through structured pruning while ensuring detection accuracy remains unaffected, and optimizing the model structure to adapt to specific edge devices (e.g., NVIDIA Jetson). Finally, our goal is to integrate the modules designed in this study into other network structures to enhance detection accuracy for a broader range of object detection tasks.

## 6. Conclusions

We introduce DRGD-YOLO, an advanced object detection methodology derived from YOLOv11n, targeting the resolution of challenges related to tiny target recognition and multi-scale object variations within SAR-based ship detection scenarios. The incorporation of a fusion architecture (CSP_DTB) that merges Transformer mechanisms with conventional CNNs results in strengthened feature learning performance, enlarged perceptual fields, and substantial reduction in information loss associated with small-scale maritime objects. Additionally, the RepGFPN neck network is utilized to optimize multi-scale feature fusion, further improving the model’s detection performance for multi-scale targets. Finally, the designed Dynamic Dual Alignment Detection Head (DYDDH) enhances the localization and classification accuracy of the detection head by facilitating information exchange between classification and localization tasks. Experimental results show that the improved DRGD-YOLO achieves an mAP50 of 93.1% and an mAP50–95 of 69.2% on the HRSID dataset, representing improvements of 3.3% and 4.6% over the original YOLOv11n algorithm, respectively. Generalization experiments on the SSDD and LS-SSDD-v1.0 datasets also validated the model’s robustness, with all metrics outperforming the baseline model. Visualization inference and heatmap experiments further demonstrated that DRGD-YOLO can more accurately focus on target features, reduce false negatives and false positives, and pay higher attention to small targets, particularly in complex backgrounds and multi-scale target scenarios. Extensive experimental results confirm that the model proposed in this paper exhibits robust performance in detecting ship targets in SAR images.

## Figures and Tables

**Table 1:** Training hyperparameters.

Hyperparameter	Value
The initial learning rate	0.01
Momentum	0.937
Total training rounds	400
Batch size	32
Size of input image	640 × 640
Optimizer used	SGD
Weight decay	0.0005
Working threads	6
Early stop rounds	50
Pre-training weights	Not loaded
Mixed Precision Used	Used

**Table 2:** Meaning of evaluation indicators.

Precision	Hidden Meaning
AP50:95	AP for IoU = 0.50:0.05:0.95
AP50	AP for IoU = 0.50
AP75	AP for IoU = 0.75

**Table 3:** HRSID dataset ablation experiments.

RepGFPN	DYDDH	CSP_DTB	P (%)	R (%)	mAP 50 (%)	mAP 75 (%)	mAP 50:95 (%)	F1 (%)	FLOPs (G)	Params (M)
			91.5	80.1	89.8	74.3	65.2	85.4	6.3	2.6
√			90.6	83.7	91.6	76.9	68.5	86.2	8.2	3.6
	√		90.6	83.9	91.9	76.0	67.2	87.1	7.9	2.2
		√	92.2	80.1	90.8	74.7	66.5	85.7	6.2	2.5
√	√		91.7	83.9	92.5	76.3	67.9	87.6	10.6	3.1
√		√	92.3	81.2	91.4	75.2	67.0	86.4	8.0	3.5
	√	√	92.1	84.0	92.5	77.2	68.2	87.9	7.7	2.1
√	√	√	**92.4**	**85.6**	**93.1**	**78.5**	**69.8**	**88.9**	10.4	3.0

Bold text indicates the best indicators in this category. “√” indicates the method used, as shown in the table. When each module is used separately, all metrics of the algorithm are improved. When two modules are used in combination, all metrics show significant improvement. Although the combination of GFPN and CSP_DTB does not bring significant improvement in most metrics, its detection accuracy (P) is improved. When all three modules are used together, all metrics in the experiment reach their maximum values.

**Table 4:** Comparative experiments with various pyramid structures.

Method	mAP50 (%)	mAP75 (%)	mAP50:95 (%)	F1 (%)	Params (M)	FLOPs (G)
Baseline	89.8	74.3	65.2	85.4	2.6	6.3
RepGFPN [23]	91.3	77.3	67.6	86.7	3.6	8.4
BIFPN [33]	90.2	75.0	65.9	85.5	1.9	6.4
GDFPN [34]	90.4	75.1	65.9	86.0	3.7	8.4
HSFPN [35]	89.9	73.1	64.9	85.1	1.8	5.7

**Table 5:** Comparative analysis of the performance of various inspection heads.

Method	mAP50 (%)	mAP75 (%)	mAP 50:95 (%)	F1 (%)	Params (M)	FLOPs (G)
Baseline	89.8	74.3	65.2	85.4	2.6	6.3
SEAMHead [36]	91.5	75.0	66.4	86.3	2.5	6.0
PGI [37]	91.4	76.9	67.7	86.8	3.6	8.8
MultiSEAMHead [38]	91.3	76.9	67.8	86.3	4.6	6.3
EfficientHead [39]	90.4	74.1	65.7	85.7	2.3	5.2
DYDDH (ours)	91.9	76.0	67.2	87.1	2.2	7.9

**Table 6:** Results of TCR parameter sensitivity analysis in the CSP_DTB module.

Model	TCR1 (Layer6)	TCR2 (Layer8)	mAP50 (%)	mAP75 (%)	mAP 50:95 (%)	F1 (%)	Params (M)	FLOPs (G)
	0.25	0.25	92.8	79.4	69.6	88.5	2.98	10.4
	0.25	0.5	93.1	78.5	69.8	88.9	2.95	10.4
Ours	0.25	0.75	92.1	77.5	68.5	88.2	2.94	10.4
	0.5	0.5	92.4	78.4	69.3	88.3	2.94	10.4
	0.75	0.5	92.4	78.1	69.0	88.0	2.94	10.4

**Table 7:** Comparison of test parameter settings.

Hyperparameter	Value
The initial learning rate	0.01
Momentum	0.937
Total training rounds	400
Batch size	32
Size of input image	640 × 640
Optimizer used	SGD
Weight decay	0.0005
Working threads	6
Early stop rounds	0
Pre-training weights	Not loaded
Mixed Precision Used	Not Used

**Table 8:** Comparative experiments on the HRSID dataset.

Method	Params (M)	FLOPs (G)	P (%)	R (%)	mAP50 (%)	mAP75 (%)	mAP50:95 (%)	F1 (%)
YOLO12n	2.55	6.0	90.0	79.5	89.2	72.9	64.4	84.4
YOLO12s	9.41	19.4	92.3	81.5	91.5	78.2	69.0	86.5
YOLO11n	2.58	6.3	91.5	80.1	89.8	74.3	65.2	85.4
YOLO11s	9.41	21.5	91.9	83.6	91.8	77.8	70.5	87.6
YOLOv10n	2.27	6.7	90.3	83.2	92.1	77.9	68.1	86.6
YOLOv10s	7.22	21.6	91.2	84.4	92.7	79.8	69.5	87.6
YOLOv9-tiny	2.62	10.6	90.2	81.3	90.4	73.2	64.9	85.5
YOLOv8n	3.01	8.7	91.7	79.7	90.2	74.4	65.4	85.3
YOLOv8s	11.13	28.6	91.0	83.4	92.1	76.7	67.8	87.0
Faster-RCNN	41.35	208	89.9	75.5	85.4	75.5	65.4	82.1
RTMDet-tiny	4.87	8.03	86.8	69.4	79.2	61.7	55.6	77.1
Cascade-RCNN	69.15	236	90.8	78.9	87.8	78.7	68.4	84.4
YOLOX-tiny	5.03	7.58	89.4	84.5	91.3	74.5	66.1	86.9
Ours	2.95	10.4	92.4	85.6	93.1	78.5	69.8	88.9

**Table 9:** Comparative experiments on the SSDD and LS-SSDD-v1.0 datasets.

Dataset	Method	mAP50 (%)	mAP75 (%)	mAP50:95 (%)	F1 (%)	FLOPs (G)	Params (M)
LS-SSDD-v1.0	YOLOv12n	74.5	14.9	29.8	74.3	6.0	2.55
	YOLOv12s	75.3	15.2	30.2	75.3	19.4	9.41
	YOLOv11n	74.1	14.5	29.7	73.8	6.3	2.58
	YOLOv11s	74.1	15.1	29.8	74.4	21.5	9.41
	YOLOv10n	71.5	15.7	29.2	70.1	6.7	2.27
	YOLOv10s	73.0	16.0	30.4	71.5	21.6	7.22
	YOLOv9-tiny	74.5	15.9	30.3	74.5	10.6	2.62
	YOLOv8n	74.6	15.6	29.9	74.4	8.7	3.01
	YOLOv8s	75.9	15.5	30.2	75.7	28.6	11.13
	Faster-RCNN	73.0	16.5	29.9	72.1	208	41.35
	RTMDet-tiny	64.5	12.2	25.7	61.7	8.03	4.87
	Cascade-RCNN	76.4	17.8	32.0	75.2	236	69.15
	YOLOX-tiny	72.4	11.8	28.0	72.6	7.58	5.03
	Ours	75.6	15.6	30.1	75.2	10.4	2.95
SSDD	YOLOv12n	97.1	87.2	71.6	93.9	6.0	2.55
	YOLOv12s	97.5	90.2	74.8	95.6	19.4	9.41
	YOLOv11n	97.5	90.2	73.2	94.7	6.3	2.58
	YOLOv11s	98.0	91.2	74.4	95.1	21.5	9.41
	YOLOv10n	97.3	88.5	72.8	94.2	6.7	2.27
	YOLOv10s	94.7	71.6	61.7	91.6	21.6	7.22
	YOLOv9-tiny	97.3	88.9	72.2	94.0	10.6	2.62
	YOLOv8n	97.1	91.4	74.0	94.9	8.7	3.01
	YOLOv8s	97.6	91.7	75.4	95.4	28.6	11.13
	Faster-RCNN	96.4	86.1	71.3	91.9	208	41.35
	RTMDet-tiny	95.8	85.1	70.3	91.5	8.03	4.87
	Cascade-RCNN	96.4	87.4	72.7	91.8	236	69.15
	YOLOX-tiny	93.7	59.6	55.1	90.6	7.58	5.03
	ours	98.2	91.9	76.8	95.9	10.4	2.95

**Table 10:** Comparison of computational efficiency and inference speed.

Method	Params (M)	FLOPs (G)	FPS
Baseline	2.55	6.3	88.7
Ours	2.95	10.4	66.7

## Data Availability

The original contributions presented in this study are included in the article. Further inquiries can be directed to the corresponding author.
